# 

**Published:** 1829-04-01

**Authors:** 


### CONTENTS

OF THE

### MEDICO-CHIRURGICAL REVIEW.

No. XX. APRIL 1, 1829.

### REVIEWS.

I.

Researches into the Causes, Nature, and Treatment of the Diseases of Warm Climates. By JAMES ANNESLEY, Esq.—[Chronic Hepatitis and Organic Disease of Liver] .. .. .. .. .. .. .. .. .. .. .. .. .. .. .. 309

II.

Cases illustrative of the Morbid Appearances which occasionally take place in the Intestines during the Progress of Fever. By RICHARD BRIGHT, M.D. .. .. 321

III.

Pathological and Practical Researches on Diseases of the Stomach, &c. &c. By JOHN ABERCROMBIE, M.D. .. .. .. .. .. .. .. .. .. .. .. .. .. .. 328

IV.

A Letter to the Secretary of State for the Home Department, on Anatomy. By G. J. GUTHRIE, Esq. .. .. .. .. .. .. .. .. .. .. .. .. .. .. .. 355

V.

An Essay on Remittent and Intermittent Diseases, &c. By Dr. MACCULLOCH.—

ART. IV. (and last) Treatment of Neuralgia .. .. .. .. .. .. .. .. 357

1. Rheumatism of the Eye, or Neuralgic Ophthalmia .. .. .. .. .. 357

2. On the Connexion between Neuralgia and Intermittent .. .. .. .. 365

3. On the Cure of Neuralgia in general .. .. .. .. .. .. .. .. 368

VI.

Observations on Polypus of the Uterus. By JOHN MACFARLANE, M.D. .. .. .. 373

VII.

Illustrations of the Diseases of the Breast. By Sir ASTLEY COOPER, Bart. &c. .. 381

VIII.

Transactions of the Medical and Physical Society of Calcutta. Vols. II. and III.. 399

ART. I. On Diarrhœa Hectica. By Mr. J. TYTLER .. .. .. .. .. .. .. 399

II. Cases of Phthisis Pulmonalis. By J. BIRD, Esq. A.M.. .. .. .. .. 401

III. On the Treatment of Indian Rheumatism. By J. HENDERSON, Esq.. 403

IV. On Sloughing Ulcers. By J. LANGSTAFF, Esq. Benares .. .. .. 404

IX.

A Treatise on Diseases of the Bones. By BENJAMIN BELL, &c. .. .. .. .. 405

X.

A Treatise on the Diseases of the Chest, and on Mediate Auscultation. By M. LAENNEC, translated by Dr. FORBES. *Third Edition* .. .. .. .. .. .. 420

XI.

Napoleon a Sainte Helene. Opinion d'un Medecin sur la Maladie de l'Empereur, et sur la Cause de sa Mort, &c. .. .. .. .. .. .. .. .. .. .. .. .. 423

XII.

On the Treatment of Abscess in the Liver. By Mr. ANNESLEY. .. .. .. .. .. 435

XIII.

An Essay on the Use of Nitrate of Silver in the Cure of Inflammation. By Mr. HIGGINBOTTOM.. .. .. .. .. .. .. .. .. .. .. .. .. .. .. .. .. 444

XIV.

Dr. WILLIAMS on Auscultation .. .. .. .. .. .. .. .. .. .. .. 450

### PERISCOPE.

Case of Pneuma-Thorax in a Medical Gentleman; with an Account of an Operation performed for its Relief; the Effects of the Operation; the Examination after Death, and Remarks on Pneuma-Thorax generally. By Dr. Johnson .. .. 478
Sheldrake on Dancing .. .. .. .. .. .. .. .. .. .. .. .. .. .. .. 489
On the Signs of Pulmonary Tubercles. By Charles Hastings, M.D. .. .. .. 501
On small Doses of Oil of Turpentine for the Cure of Sciatica and other Neuralgiæ. 505
On the Form of the Face and Forehead; with a Theory of their Formation in different Ages and Nations. By Dr. Milligan .. .. .. .. .. .. .. .. .. 506
Aneurismal Dilatation of the Posterior Auris and Temporal Arteries. By Mr. Syme; with Criticisms .. .. .. .. .. .. .. .. .. .. .. .. .. 507
Pathology of Muscæ Volitantes .. .. .. .. .. .. .. .. .. .. .. .. .. 512
On the Nature of Tubercles—Phthisis Pulmonalis .. .. .. .. .. .. .. .. 513
Pulmonary Apoplexy .. .. .. .. .. .. .. .. .. .. .. .. .. .. .. 514
On Abscess of the Liver .. .. .. .. .. .. .. .. .. .. .. .. .. .. 515
Imperforate Anus—Operation, &c. .. .. .. .. .. .. .. .. .. .. .. .. 515
Cases of Foreign Bodies in the Larynx and Trachea. By Dr. Reiche .. .. .. 517
On the Curative Effects of Carriage and Travelling Exercise. By M. Coriolis; with Observations by Dr. Johnson .. .. .. .. .. .. .. .. .. .. .. .. 519
Excision of the Clavicle for Osteo-Sarcoma. By Dr. Valentine Mott. .. .. .. 549
Extensive Suppuration, and Death, from the Prick of a Pin. By Mr. Cunnaghan 552
Case of Neuralgia of the Par Vagum imitating Hysteria. By Dr. Prus. .. .. 553
Sheldrake on the "Personal Appearance" of Young Ladies .. .. .. .. .. 554
Remarkable Alteration in the Structure of the Patella. By Dr. Knox .. .. .. 556
Amputation of the Lower Jaw. By Dr. Anderson .. .. .. .. .. .. .. .. 557
On the Respiration of Cold Air in Pulmonary Diseases. By Dr. Drake. .. .. 559
On Mesmerism; or Animal Magnetism .. .. .. .. .. .. .. .. .. .. .. 560
On Intermissions and Irregularities of the Pulse, as Caused by the Imperfections of the Mitral Valves .. .. .. .. .. .. .. .. .. .. .. .. .. 562
The Gibraltar Epidemic .. .. .. .. .. .. .. .. .. .. .. .. .. .. 564
Dr. Kennedy on the Management of Blisters .. .. .. .. .. .. .. .. .. 564

### CLINICAL REVIEW.

### Hôtel Dieu.

1. Operation of Lithotomy—Dupuytren unsuccessful .. .. .. .. .. .. .. 453
2. Calculus—Lithontripteur—Operation .. .. .. .. .. .. .. .. .. .. .. 454
3. Spontaneous Luxation of Cervical Vertebræ.. .. .. .. .. .. .. .. .. 455
4. Latent Tubercular Phthisis—Pneuma-Thorax .. .. .. .. .. .. .. .. 456
5. Varieties of Hydrocele .. .. .. .. .. .. .. .. .. .. .. .. .. .. 539
6. Suicidal Amputation of Penis .. .. .. .. .. .. .. .. .. .. .. .. 539
7. Aneurism of the Brachial Artery, &c. .. .. .. .. .. .. .. .. .. .. 540
8. Polypi and Foreign Bodies in the Meatus Auditorius .. .. .. .. .. .. 573
9. Angina Pectoris.. .. .. .. .. .. .. .. .. .. .. .. .. .. .. .. 573
10. Operation for Femoral Hernia—Artificial Anus Cured .. .. .. .. .. .. 574
11. Varix of the Saphena imitating Hernia.. .. .. .. .. .. .. .. .. .. 575

### Glasgow Royal Infirmary.

1. Synovitis .. .. .. .. .. .. .. .. .. .. .. .. .. .. .. .. .. 457

2. Excision of Part of the Tunica Vaginalis, &c. .. .. .. .. .. .. .. .. 457

3. Purulent Depôts in the Knee .. 458
4. Singular Case of Emphysema .. 533
5. Compound Fracture of the Cranium .. 586
6. Taliacotian Operation .. 588
7. Fungous Tumours from the Gums .. 588
8. Hospital Gangrene .. 589

New York Hospital.

1. On the Mercurial Treatment of Fever. By Dr. Brown .. 459

La Charite.

1. Epilepsy of 14 Years' standing cured .. 460
2. Remarkable Succession of Morbid Phenomena .. 460
3. Ascites cured by Perspiration .. 461
4. Acute Rheumatism—Antimonial Treatment .. 461
5. Anomalous Disorder imitating Organic Diseases .. 461
6. Spinal Disease .. 462
7. Wound of the Heart .. 463
8. Phlegmatia Dolens in a Male .. 526
9. Hæmorrhoids—Mode of operating at the above Hospital .. 527
10. On Hypertrophy of the Brain; with Cases, Dissections, and Observations. By M. Laennec, M.D. .. 565
11. Partial Dislocation of the knee .. 582
12. Caries of the First and Second Cervical Vertebræ—Dislocation of the Atlas .. 582

St. Thomas's Hospital.

1. Ulceration of the Scrotum—Fungus of Testicle .. 465
2. Lithotomy .. 465
3. Purulent Ophthalmia succeeding to Gonorrhœa .. 528
4. Extraordinary and alarming Effects of drinking Porter after severe Exercise .. 530
5. Taliacotian Operation Successful .. 571

Hospital of Nantes.

1. Angina Pellicularis .. 466
2. Œdema of the Glottis .. 467
3. Ovarian Tumour cured .. 467
4. Trismus Traumaticus cured .. 468
5. General Phlebitis .. 468

Worcester Infirmary,

1. Dislocation into the Ischiatic Notch .. 469

2. Aneurism of the posterior Tibial Artery .. 469

3. Tumour on the Orbit—Abscess in the Brain .. 470

Hôpital des Veneriens.

1. On the Influence of Mercury on the Functions of the Uterus. By Dr. Alexander Colson .. 471

Guy's Hospital.

1. Scirrhus of the Human Male Breast .. 472

2. Non-union of Fractured Humerus. By Mr. Amesbury. .. 563

### Westminster Ophthalmic Hospital.

1. Report on the Treatment of Cataract .. .. .. .. .. .. .. .. .. .. .. .. .. 472

### Hitchin Dispensary.

1. Suicidal and Infanticidal Mania .. .. .. .. .. .. .. .. .. .. .. .. .. 474

### Westminster Hospital.

1. On Hernia .. .. .. .. .. .. .. .. .. .. .. .. .. .. .. .. .. 475
On Amputation .. .. .. .. .. .. .. .. .. .. .. .. .. .. .. .. 477

### St. George's Hospital.

1. Loose Cartilages in the Knee-joint—Operation attempted without Success .. 524
2. Fracture of the Neck of the Thigh-bone—Delirium Traumaticum—Opium .. 525
3. Case of Suicidal Cut-throat, with curious circumstances .. .. .. .. .. .. 575
4. Insidious and Fatal Pleuritis after a trifling operation .. .. .. .. .. .. 577
5. Ranula—Treatment by Seton .. .. .. .. .. .. .. .. .. .. .. .. .. 578
6. Erysipelas .. .. .. .. .. .. .. .. .. .. .. .. .. .. .. .. .. 579
7. Important Case of Wound of the Femoral Artery and Vein in the Sheath of the Triceps—Gangrene of the Limb—Death .. .. .. .. .. .. .. .. .. 589
8. Parallel Case at Haslar Hospital .. .. .. .. .. .. .. .. .. .. .. .. 593
9. Commentaries .. .. .. .. .. .. .. .. .. .. .. .. .. .. .. .. 594

### Hôpital de Beaucaire.

1 Acute Intermittent Dyspnœa .. .. .. .. .. .. .. .. .. .. .. .. .. .. 532

### Hôpital Beaujon.

1. Contusion of the Perineum—Fracture and Separation of the Pelvis.. .. .. .. 534
2. Canceroid Ulcer of the Upper Lip .. .. .. .. .. .. .. .. .. .. .. .. 535
3. High operation for the Stone Unsuccessful—Recto-vesical Operation resorted to 569

### Gloucester Infirmary.

1. Dislocation of the Femur into the Ischiatic Notch .. .. .. .. .. .. .. 537
2. Large Lacerated Wound of the Perineum .. .. .. .. .. .. .. .. .. .. 538
3. Difficult Case of Lithotomy .. .. .. .. .. .. .. .. .. .. .. .. .. 538

### Hopital de Versailles.

1. Ulcerated Cancer of the Rectum cured by Extirpation .. .. .. .. .. .. 581

### Hotel Dieu—Cochin—La Pitie.

1. Researches on Chronic Inflammation of the Cerebral Vessels .. .. .. .. .. 585

### MEDICAL JURISPRUDENCE,

### Medical Societies—London and Westminster.

1. Expulsion of Mr. Lambert, with Reflections and Observations .. .. .. .. .. 493
2. Medical Justice and Liberality—New Pathological Phenomenon .. .. .. .. 498
3. Professional or unprofessional? that is the Question! Mr. Wakley's Report of the late Trial.. .. .. .. .. .. .. .. .. .. .. .. .. .. .. .. .. 499
4. Surgical Reform.. .. .. .. .. .. .. .. .. .. .. .. .. .. .. .. .. 500
5. Medical Reform in France—Convocation general, of Physicians and Surgeons for the purpose of devising a Plan of Medical Education and Police.. .. .. 542
6. On the Consequences of Reporting from Medical Societies .. .. .. .. .. 543
7. On Infirmaries and Dispensaries .. .. .. .. .. .. .. .. .. .. .. .. 544
8. Hospital Reports—Hospital Elections: Sir. A. Carlisle's Letter—Dissentions in the Surgical Cabinet.. .. .. .. .. .. .. .. .. .. .. .. .. .. .. .. .. 546
Bibliographical Record .. .. .. .. .. .. .. .. .. .. .. .. .. .. .. .. .. 595

